# The effects of ARID1A mutation in gastric cancer and its significance for treatment

**DOI:** 10.1186/s12935-023-03154-8

**Published:** 2023-11-26

**Authors:** Shan Lu, Ruifeng Duan, Liang Cong, Ying Song

**Affiliations:** Gastroenteric Medicine and Digestive Endoscopy Center, The Second Hospital of Jilin University, Changchun, China

**Keywords:** ARID1A mutation, Gastric cancer, Prognosis, Treatment, Biomarker

## Abstract

Gastric cancer (GC) has emerged as a significant issue in public health all worldwide as a result of its high mortality rate and dismal prognosis. AT-rich interactive domain 1 A (ARID1A) is a vital component of the switch/sucrose-non-fermentable (SWI/SNF) chromatin remodeling complex, and ARID1A mutations occur in various tumors, leading to protein loss and decreased expression; it then affects the tumor biological behavior or prognosis. More significantly, ARID1A mutations will likely be biological markers for immune checkpoint blockade (ICB) treatment and selective targeted therapy. To provide theoretical support for future research on the stratification of individuals with gastric cancer with ARID1A as a biomarker to achieve precision therapy, we have focused on the clinical significance, predictive value, underlying mechanisms, and possible treatment strategies for ARID1A mutations in gastric cancer in this review.

## Introduction

Gastric cancer (GC) is a disease that affects individuals worldwide. In 2021, there were reportedly 1.1 million new cases, and diagnosing stomach carcinoma at an advanced stage resulted in high mortality, a high recurrence rate, and a bad prognosis [1]. Research has confirmed that various etiologies play a crucial part in the pathogenesis of GC, such as microbiota, smoking, alcohol consumption, family genetic factors, previous gastric surgery, and poor eating habits. With the rapid development of research in molecular biology and the ongoing maturation of sequencing technology, the connection between classical genetics and epigenetics and stomach cancer has received attention. The research directions mainly include gene mutation, gene deletion, DNA methylation, histone modification, and chromatin remodeling. AT-rich interactive domain 1 A (ARID1A) genes usually have inactive mutations. Clear cell carcinoma (40–57%), GC (8–27%), bladder cancer (about 20%), hepatocellular carcinoma (10–17%), melanoma (about 12%), colon cancer (about 9%), and lung cancer (about 8%) are a few varieties of cancer that show a high incidence of mutations that render ARID1A inactive with loss of expression [2–9]. Various cancer treatment options associated with ARID1A mutations are undergoing clinical trials; they have been listed in Table 1. The clinical importance and molecular mechanism of the ARID1A mutation in GC and its role in traditional targeted treatment and emerging immune checkpoint blockade (ICB) therapy have not been thoroughly investigated. From molecular mechanisms to potential clinical treatment strategies, this article comprehensively reviews the functionality of ARID1A mutation in GC, especially its possible correlation with other biological molecules in tumorigenesis and its predictive value in treatment selection and prognosis. Studying the function and role of ARID1A in GC carcinogenesis will help to improve patients’ prognoses and guide clinical practice.

**Table 1:** Clinical trials for cancer therapies involving ARID1A

Number	Study types	Status	Phase	Cancer types	Intervention	Primary endpoint
NCT04065269	Interventional (Clinical Trial)	Recruiting	II	Gynecological Cancers	ATR inhibitor (AZD6738), PARP inhibitor (Olaparib)	ORR
NCT04957615	Interventional (Clinical Trial)	Recruiting	II	Metastatic Malignant Solid Neoplasm, Unresectable Solid Neoplasm	Nivolumab	ORR, OS
NCT05523440	Interventional (Clinical Trial)	Recruiting	II	Recurrent Endometrial Carcinoma Recurrent Ovarian Carcinoma	Bevacizumab, Niraparib	ORR
NCT05690035	Interventional (Clinical Trial)	Not yet recruiting	II	Metastatic Colorectal Cancer	Tislelizumab & Fruquintinib	ORR
NCT04953104	Interventional (Clinical Trial)	Not yet recruiting	II	Urologic Neoplasms	Nivolumab	ORR, OS
NCT03682289	Interventional (Clinical Trial)	Recruiting	II	Solid Tumors	Ceralasertib, Olaparib, Durvalumab	ORR
NCT04042831	Interventional (Clinical Trial)	Recruiting	II	Advanced Biliary Tract Cancer	Olaparib	ORR
NCT04284202	Interventional (Clinical Trial)	Unknown	II	NSCLC Stage IV	PD-1 plus Dasatinib	PFS

ATR: ataxia telangiectasia and rad3-related; PARP: poly (ADP-ribose) polymerase; PD-1: Programmed cell death protein 1; ORR: Objective Response Rate; OS: overall survival; PFS: Progress Free Survival

### Chromatin remodeling and chromatin remodeling complexes

**Fig. 1:**
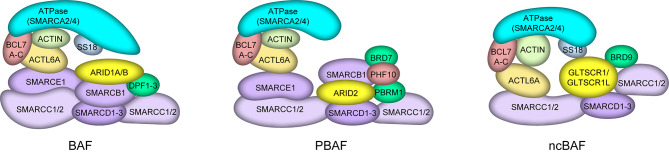
The structure of three types of mammalian switch/sucrose-non-fermentable complexes (BAF, PBAF, and ncBAF).

As the basic structural unit of chromatin, the nucleosome consists of an octamer of histones and double-stranded DNA surrounded by negative supercoils that are highly concentrated to produce a dense structure. Dynamic alterations such as histone insertion, expulsion, and nucleosome sliding cause changes in the spatial location of histones and DNA, making it simpler for protein regulators to approach double-stranded DNA to complete DNA replication, transcription, recombination, and other processes. This is known as chromatin remodeling [10]. The energy released by ATP hydrolysis is used by the ATP-dependent chromatin remodeling complex to drive chromatin conformational changes and control gene expression. The ATPase subunit, which belongs to the SF2 helicase family, is the most important protein component in the ATP-dependent chromatin remodeling complex. Based on their domains, ATPase subunits are divided into four subfamilies: SWI/SNF, INO80, ISWI, and CHD [11]. The switch/sucrose-non-fermentable (SWI/SNF) chromatin remodeling complex was first discovered in *S. cerevisiae* in the 1990s. It is a transcriptional regulatory complex composed of multiple genes encoding proteins. In mammalian cells, three types of SWI/SNF complexes have been thoroughly investigated: canonical BAF (CBAF) containing accessory subunits ARID1A/ARID1B and DPF1/2/3, polybromo-associated BAF (PBAF) with ARID2, PHF10, PBRM1, and BRD7 as marker subunits and non-canonical BAF (ncBAF) with BRD9 and GLTSCR1 or GLTSCR1L (GLTSCR1-like) as its distinct subunit. They all include the ATPase catalytic subunit SMARCA4 or SMARCA2, and they all also have individual subunits that make different complexes unique [12–16] (Fig. 1).

### Structure and function of ARID1A

#### Structure of ARID1A

**Fig. 2:**
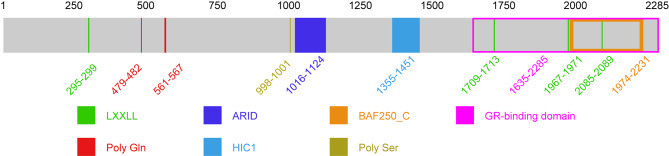
The structure of AT-rich interactive domain 1 A

ARID1A, also named as OSA1, P270, hOSA1, BAF250, C1orf4, BAF250a, and SMARCF1, is a SWI/SNF complex component. The ARID1A gene, which codes for a protein with 2285 amino acid residues and a relative molecular mass of 240 KD, is found on chromosome 1p36.11. ARID1A, typically located in the nucleus, is strongly expressed in various body tissues [17]. ARID1A protein mainly contains a conserved domain (aa 1016–1124) that binds to adenine (A) and thymine (T)-rich DNA sequences, a HIC1-binding domain (aa 1355–1451), a glucocorticoid receptor (GR) binding domain (aa 1635–2285), and four LXXLL motif structures [18, 19] (Fig. 2). ARID1B has a conserved domain that is highly similar to ARID1A. Their expression in development and cell regulation differs, resulting in different biological functions in vivo [20]. In recent years, ARID1A and ARID1B co-mutations have been found in various cancers, but at least one functional allele has survived. A recent study found that their double deletion may lead to the redistribution of the cBAF complex after division and may affect the oligomerization of PBAF [21]. In ARID1A mutant tumor cells, the loss of ARID1B leads to the loss of enhancer structure and changes in chromatin accessibility, making it challenging for cancer cells to survive. Similar to the interdependence of SMARCA4 or SMARCA2, the interplay between ARID1A and ARID1B synthetic lethality in oncology needs to be further studied [22–25].

### Biological function of ARID1A

#### ARID1A and cell stemness

ARID1A plays an outstanding role in regulating the differentiation of a wide assortment of stem cells, including cardiac progenitor cells, neural stem/progenitor cells, as well as embryonic stem cells. In the mouse model, by altering the accessibility of chromatin, ARID1A controls the expression of crucial genes during myocardial development, promoting the differentiation of cardiac progenitor cells into normal cardiomyocytes [26]. ARID1A gene knockout inhibits the self-renewal characteristics of embryonic stem cells, and mesoderm differentiation is negatively impacted, seriously impeding embryonic development [27]. Liu et al. used Cre/loxP to construct ARID1A neural stem cell conditional knockout mice, demonstrating that the absence of ARID1A function impairs radial glial cell proliferation and leads to the dysregulation of genes related to neural stem progenitor cell differentiation, for instance, Fezf2, Rgs6, Ptk2b, and Lpar1 [28]. According to Wang et al., the lack of ARID1A causes the destruction of the structure and function of the SWI/SNF complex, which in turn leads to an imbalance in the expression of genes involved in cell stemness, cell differentiation, and liver function, which promotes the growth of liver cancer [29]. Meanwhile, research has demonstrated that ARID1A is crucial for preserving the functionality of pancreatic acinar cells and re-proliferation following damage. The differentiation of ARID1A-deficient pancreatic tumors is blocked and they are endowed with high mobility, invasiveness, and stem-like properties [30].

#### ARID1A and DNA damage repair

DNA is vulnerable to various endogenous and exogenous factors, resulting in changes in genetic information carriers, leading to major diseases such as cancer. The DNA damage response (DDR) allows organisms to sense DNA damage signals, slow down or block cell cycle progression, and activate different DNA repair mechanisms or apoptosis mechanisms. DNA double-strand break (DSB) is a common form of DNA damage. In mammalian cells, DSB has two common repair pathways: non-homologous end joining (NHEJ), which occurs mainly in the S phase of the cell, and homologous recombination (HR), which occurs primarily in the G1 and G2 phases. The balanced development of the two repair pathways keeps the genome stable. Recent findings have pointed out that ARID1A is essential in the two DNA damage repair pathways. The recruitment of the SWI/SNF complex ATPase subunit to the DNA damage site depends on the presence of ARID1A. Inhibition of ARID1A reduces the accumulation of NHEJ pathway initiator KU70/KU80 in DNA DSB, leading to the inactivation of the NHEJ pathway [31]. Ataxia telangiectasia mutant gene (ATM) and ataxia telangiectasia andrad3-related (ATR), both members of the PI3/PI4 kinase family, are essential for the HR-mediated DSB response. ARID1A is recruited to DNA DSBs and interacts with the vital kinase ATR. As a result, the cell cycle is stopped, and broken DNA repair is facilitated [32].

#### ARID1A and tumor cell proliferation, invasion, metastasis, and apoptosis

ARID1A is generally considered a tumor suppressor gene that can inhibit the biological behavior of malignant tumors and regulate the cell cycle to promote apoptosis to exert anticancer effects. He et al. [33] discovered that human hepatocellular carcinoma (HCC) cell lines Huh-7 and MHCC-97 H express ARID1A differently. The former was relatively high, and the latter was deficient. ShRNA-mediated ARID1A knockdown significantly promoted the migration and invasion of Huh-7 cells. On the contrary, overexpressing ARID1A markedly inhibited the ability of MHCC-97 H cells to invade and migrate. In addition, in vivo experiments of mouse xenograft tumors showed that lung metastasis occurred in HCC cells knocking down ARID1A in 50% (3/6) of mice, demonstrating that a decrease in ARID1A expression was related to HCC metastasis.

Angiogenesis is an essential process of tumor growth, invasion, and metastasis. Yoodee et al. [34] found that after knocking down ARID1A in colorectal cancer cell line Caco-2, the secretion level of the angiogenic factor VEGF was significantly increased by ELISA. A previous study by his team also showed that the human endothelial cells’ down-regulation of ARID1A promotes Ang2 secretion and endothelial cell activity, which induces angiogenesis [35].

Apoptosis is the term used to describe spontaneous and planned cell death, which is regulated by genes to preserve the stability of the internal environment. Some researchers have shown that the knockdown of ARID1A in leukemia cell lines can resist FAS-mediated apoptosis [36]. Zhang et al. [37] found that siRNA knockdown of ARID1A significantly increased the expression of cyclin D1, Bcl-2, and Akt phosphorylation and inhibited paclitaxel-induced apoptosis. Xie et al. [38] found that the depletion of ARID1A promoted the proliferation of colorectal cancer cell lines and inhibited 5-fluorouracil-induced apoptosis. In recent years, researchers have shown that impaired ARID1A expression in GC cells may resist Harakiri-mediated apoptosis and lead to disease [39]. These findings demonstrate the role of ARID1A in apoptosis.

It has been found that the absence of ARID1A may lead to changes in EMT markers. Tomihara et al. [40] used pancreatic ductal adenocarcinoma cell lines to study the interaction between ARID1A and EMT regulatory proteins. After ARID1A knockout, the levels of epithelial markers cytokeratin-19 and E-cadherin decreased, and the level of cytoplasmic marker vimentin increased. Somsuan et al. [41] found that siARID1A transfection increased the levels of renal interstitial markers (fibronectin and vimentin) and decreased the levels of epithelial markers (E-cadherin and ZO-1) when they examined the effect of ARID1A expression reduction on the characteristics of non-malignant renal cell carcinogenesis. This is likely to be related to the TGF-b1/SNAI1 signaling pathway. The same results were obtained in the malignant renal cell carcinoma cell line 786-O. According to these studies, a lack of ARID1A could trigger EMT and promote tumor cell metastasis.

### Association between GC and ARID1A mutation or ARID1A protein expression loss

#### Relationship between GC subtypes and ARID1A mutation

GuanBin’s analysis of 257 cases of somatic ARID1A mutation data showed that ARID1A mutations were mostly frame-shift mutations or nonsense mutations. Nonsense-mediated RNA decay (NMD) or incorrect protein degradation leads to abnormal expression of ARID1A protein, ultimately leading to tumor inhibition loss [42]. Despite the fact that ARID1A mutation is linked to loss of expression, the gene mutation is not the only cause of the loss of protein expression. Recently, it was discovered that ubiquitination, followed by proteasomal degradation, was the cause of the ARID1A protein’s disappearance in GC cells [43]. ARID1A, the second-largest mutant gene after TP53 in GC, can detect mutations in 8–27% of GC cases [4–9, 44].

Interestingly, the frequency of ARID1A mutations differs significantly in different subtypes of GC. The Cancer Genome Atlas (TCGA) conducted complex statistics and informatics analysis on 295 cases of GC tissue and blood samples and formally divided GC into four molecular subtypes, including Epstein-Barr virus (EBV)-positive, microsatellite instability (MSI), chromosome instability (CIN), and genomic stability (GS). Frequent ARID1A mutations were found in GC that was EBV-positive [45]. In 2015, GC was reclassified into four subtypes by the Asian Cancer Research Group (ACRG) to better direct treatment and prognosis: MSI, MSS / EMT, MSS / TP53 +, and MSS / TP53-. The mutation rates of ARID1A were 19/43 (44.2%), 5/36 (13.9%), 11/59 (18.6%), and 5/85 (5.9%), respectively [46].

Mechanistically, the deletion of ARID1A significantly increased the efficiency of EBV infection in gastric epithelial cells and it was challenging to recruit mismatch repair proteins, which initiated the occurrence of EBV subtype and MSI subtype GC [47, 48]. Setia et al. used immunohistochemistry and in situ hybridization to classify GC more easily: EBV-positive, MSI-H, aberrant expression of E-cadherin, aberrant expression of P53, and normal expression of P53. It was found that EBV-positive and MSI-H (high microsatellite instability) gastric cancer had a better prognosis [49]. Subsequently, according to the above classification method, some researchers found that the loss of ARID1A protein expression in GC was significantly related to the positive expression of the MSI-H subtype and PD-L1 [50]. Since gastric cancer with the MSI-H subtype and PD-L1 positive expression more effectively responds to immune checkpoint inhibitors (ICIs), the expression of ARID1A may become a biomarker for GC immunotherapy [51].

#### Clinical and prognostic relevance of ARID1A mutation or protein loss in GC

A study that divided up GC tissue samples in accordance with the degree of ARID1A expression revealed that either total or partial loss of ARID1A expression was linked to a shorter progression-free survival (PFS) and overall survival (OS) [52]. Another study found a significant correlation between the deletion of ARID1A and tumor differentiation (P = 0.009), metastasis to lymph nodes (P = 0.030), and tumor size (P = 0.022) [9]. Zhou et al. [53] discovered that the expression level of ARID1A protein in GC tissues was significantly lower than in the normal tissues adjacent to the GC tissues. The expression level of ARID1A was interrelated with the depth of tumor invasion (P = 0.040). In line with this, Wang et al. [51] ascertained that ARID1A protein deletion is an independent risk factor for the poor prognosis of GC after analyzing 272 primary GC samples by Immunohistochemistry (IHC) and quantitative reverse transcription PCR (qRT-PCR). These findings demonstrate, from several angles, that the absence of ARID1A protein expression is related to a poor prognosis for GC.

However, not all studies support this view. For example, Ibarrola-Villava et al. [54] found that compared to patients with ARID1A positive expression, the OS of patients with ARID1A negative expression was considerably higher (P = 0.03). In a cohort study using tissue microarray technology (n = 173), There was no discernible link between OS and loss of ARID1A expression [55]. Although there is some controversy, a meta-analysis related to GC and ARID1A support that the decrease in ARID1A expression is connected to adverse clinical outcomes [56].

The following factors may be responsible for the disagreement regarding the relationship between the expression level of ARID1A and the prognosis of GC: (1) The individual heterogeneity of GC is due to the pathological features of GC being affected by a variety of genetic and environmental factors, and thus the molecular and morphological heterogeneity of GC is formed. (2) The limitation of the number of patients may lead to limited experimental results. (3) In the process of immunohistochemical staining, due to the lack of sensitivity of the detection method, a negative reaction is caused, or the differences in criteria for the results of immunohistochemical staining and the different reagents lead to differing experimental results. These elements might be the leading causes of the differing findings among many studies. At the same time, it is worth noting that ARID1A is not the only factor affecting the prognosis of GC. Many other factors may lead to changes in the clinical prognosis of GC, such as different GC subtypes. In short, ARID1A mutation or loss of expression may result in a worsening of the biological behavior of GC, indicating that ARID1A expression level may be an essential determinant in judging the prognosis of GC. However, many experiments are still needed to confirm this.

#### Interaction between ARID1A mutation or protein deletion and other genes and gene pathways in GC

It is generally known that the TP53 gene acts as a tumor suppressor gene. The P53 protein translated by the TP53 gene can block cell cycle progression, repair damaged DNA, and promote apoptosis. It has been found that the expression of two downstream target genes of P53 decreased after ARID1A silencing in GC cells, indicating that ARID1A and P53 may synergistically activate the transcription of target genes and inhibit tumor growth [57], which confirmed the previous view of Guan in ovarian cancer research [58]. Further studies have found that ARID1A mutation or absence of ARID1A protein expression in GC negatively correlates with TP53 mutation [59]. Recently, Loe et al. [60] established a clinically applicable gastric tumor model with ARID1A loss of heterozygosity. After in-depth analysis and in vivo verification, they found that the lack of ARID1A heterozygosity in GC will lead to the extensive loss of H3K27ac modification in the enhancer region of genes related to TP53 tumor suppressor pathway and apoptosis pathway, resulting in the inhibition of p53 apoptosis-related gene expression, thereby promoting tumor progression. In addition, the team further found that activation of the TP53 signal pathway may possess a therapeutic impact on AIRD1A heterozygous GC.

The PIK3CA gene, which codes for 1068 amino acids, is found on chromosome 3q26.3. It is a catalytic subunit of the IA-type PI3Ks family. PIK3CA gene mutation activates the PI3K/AKT signaling pathway, leading to tumorigenesis. Zhang et al. [57] found that knockout of ARID1A in GC cell lines directly targets PDK1 and PIK3CA transcription in the PIK3/AKT pathway, resulting in phosphorylation changes in the main components of the PIK3/AKT signaling path, including AKT, mammalian target of rapamycin (mTOR),and glycogen synthase kinase 3α/β (GSK3α/β), among others. In this process, the HIC1 binding domain in ARID1A may play a crucial part. A similar in vitro study also confirmed that in ARID1A-deficient GC cells, the PI3K/AKT pathway was activated, which induced the proliferation of GC cells, and ARID1A-deficient GC cells were more sensitive to PI3K and AKT inhibitors [61]. Despite the low number of clinical studies, it seems that the loss of ARID1A expression can be used as a biomarker for AKT pathway activation and predict the effect of AKT inhibitors in patients with GC. Yang et al. [62] found that miR-233-3P can stimulate GC cell growth and migration by specifically targeting ARID1A, and the NF-κB/miR-223-3p/ARID1A pathway is an essential pathway for HP-mediated chronic gastritis to GC transformation.

### Potential therapeutic strategies for ARID1A and GC

#### ARID1A and immunotherapy of GC

GC is traditionally treated with surgery, radiotherapy, and chemotherapy. With the advancement of medical technology, targeted therapy, immunotherapy, angiogenesis therapy, and other new treatment methods for GC have emerged as research hotspots. It is a widely held belief that ICIs are effective in treating GC, and the clinical trials of PD-1/PD-L1 inhibitors have garnered much interest recently.

ATTRACTION-2 is a phase III multicenter trial that evaluates nivolumab versus placebo in treating patients with advanced GC who progressed after ≥ 2 lines of chemotherapy. Regarding OS and PFS, the results showed that nivolumab significantly outperformed the placebo [63]. Subsequently, as a result of the findings of the CheckMate-649 study, the FDA granted approval for the combination of nivolumab and chemotherapy in 2021, making it the first immunotherapy treatment to be authorized as a first-line treatment for GC anywhere in the world [64].

Despite the fact that ICB therapy has changed the treatment strategy for malignant tumors, a significant number of GC patients do not respond well to immunotherapy. Therefore, it is urgent to further screen out relevant biomarkers to choose patients who could gain from ICBs.

The KETNOTE-059 test showed that pembrolizumab had a more favorable therapeutic impact on gastric or esophagogastric junction (EGJ) adenocarcinoma with a PD-L1 combined positive score (CPS) ≥ 1 [65]. CHECKMATE-649 also showed that in advanced GC (AGC) and EGJ cancers, nivolumab combined with chemotherapy resulted in better OS than chemotherapy alone, especially when PD-L1 is combined with CPS ≥ 5 [64]. These critical findings highlight the importance of PD-L1 expression levels in determining how ICIs should be used.

The tumor mutation burden (TMB) can be used to assess the extent and capacity of a tumor to produce new antigens and predict how well immunotherapy will work for various tumor types. Wang et al. [66] found that TMB was connected to significant survival benefits of AGC using whole exome sequencing. The OS of the high TMB group was significantly better than that of the low TMB group (14.6 vs. 4.0 months, HR = 0.48, P = 0.038). It was confirmed that the TMB level might be a predictive biomarker for screening the survival benefit population of ICI toripalimab in treating AGC.

Different mismatch repairs (dMMRs) are usually caused by mutations in the gene encoding mismatch repair proteins. If there is a problem with the mismatch repair system, the length of the short tandem repeats changes to form microsatellite instability (MSI-H). Patients with MSI-H/dMMR have severe defects in the tumor DNA repair mechanism, but numerous studies, including KEYNOTE-016, 164, 012, 028, and 158, have demonstrated that patients with MSI-H / dMMR tumors have better immunotherapy effects. The FDA currently approves Pembrolizumab to treat patients with metastatic or unresectable solid tumors carrying dMMR or MSI-H biomarkers [67].

Many studies have confirmed that the degree of tumor-infiltrating Lymphocytes (TILs) can serve as a biomarker to forecast the favorable outcome of PD-1/PD-L1 immunosuppressive therapy by detecting the subgroup, number, and regional distribution of TILs [68, 69]. Based on three systemic inflammatory markers, Formica et al. established the Gastric Inflammation Prognostic Index (GIPI) to evaluate the prognosis of patients with a metastatic gastro-esophageal junction (mGOJ)/GC (GC) after ICI treatment [70]. Surprisingly, the expression of ARID1A in GC is intimately connected to these biomarkers that affect immune blockade therapy. The positive expression of PD-L1 in tumors is closely correlated with the loss of ARID1A in GC. In terms of the mechanism, ARID1A deficiency up-regulates PD-L1 expression by activating the PI3K/AKT/mTOR pathway [71]. In addition, researchers used bioinformatics methods to find that the increased immune activity of gastrointestinal cancer with ARID1A mutation is attributed to higher TMB levels and has better advantages in immunotherapy [72]. It has been reported that ARID1A deletion cannot recruit mismatch repair (MMR) protein MSH2 during DNA replication, resulting in increased tumor MSI subtypes and TMB levels [48]. Wang et al. [51] found that the loss of ARID1A protein expression in GC is related to dMMR status, systemic inflammatory markers, and increased PD-L1 expression levels. A study found that the expression level of ARID1A protein in early-onset GC tissues and normal mucosal tissues around the tumor was associated with tumor T-cell infiltration [73].

A recent study discovered that the SWI/SNF complex gene (especially the ARID1A gene) has a high mutation rate in various cancers and is associated with high TMB status and MSI subtypes. After ICI treatment for malignant tumors, the three significant tumor treatment endpoint indicators (progression-free survival [PFS], overall response rate [ORR], and disease control rate [DCR]) were significantly prolonged [74]. The discovery of a link between ARID1A inactivation and PD-L1, TMB, MMR, TILs, and systemic inflammatory markers in GC raises the possibility that ARID1A deletion might serve as a predictive biomarker for ICBs therapy for GC. More research is needed to understand the underlying mechanisms of the correlation between immunotherapy biomarkers so that patients with malignant tumors have a better chance of survival after receiving ICBs treatment.

#### Other treatments for ARID1A-deficient GC

**Fig. 3:**
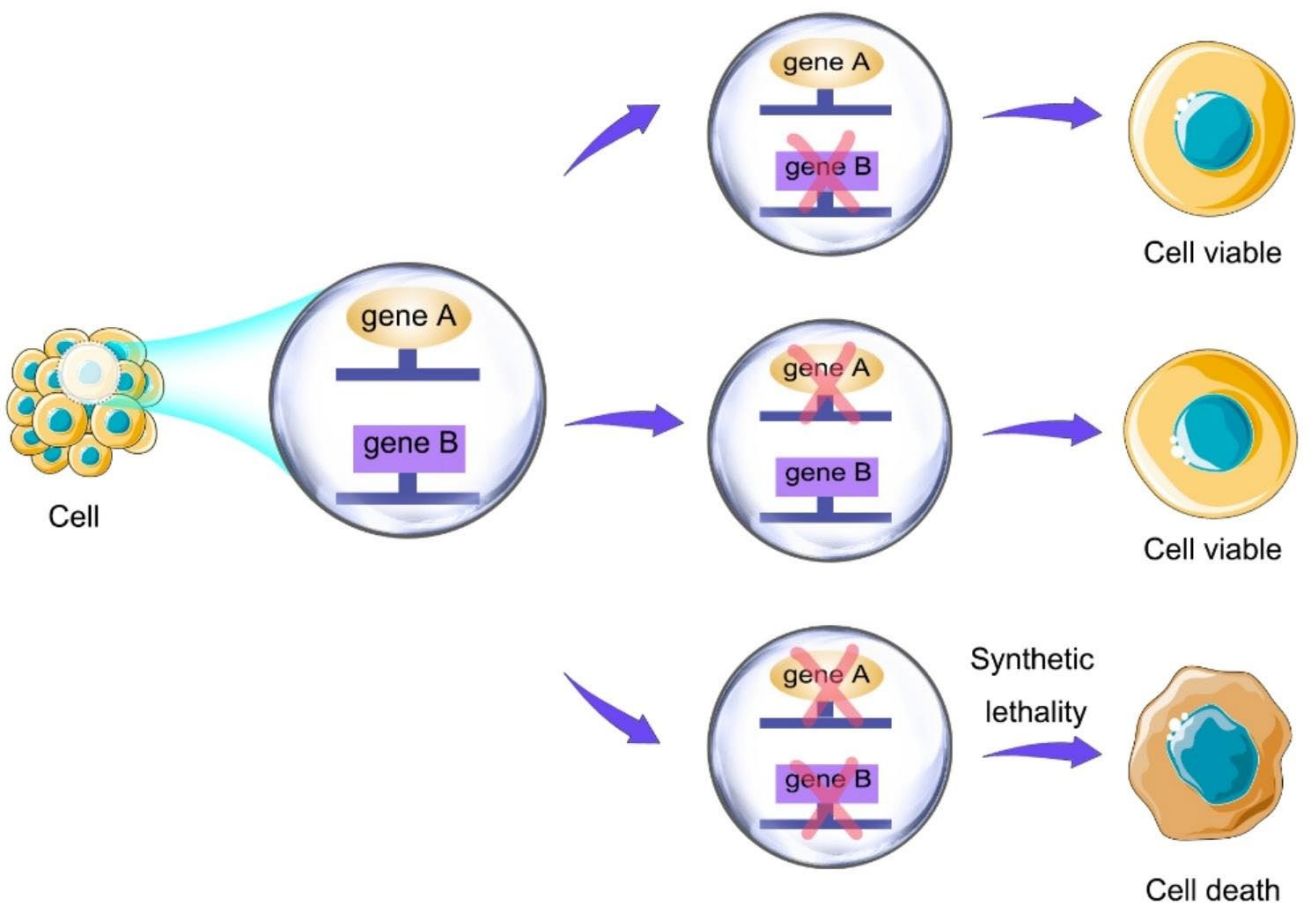
Basic principles of synthetic lethal therapy. When gene A and gene B exist at the same time, the cells survive. If one of gene A and gene B is absent, the cells can survive. If gene A and gene B lose expression at the same time, cell death will occur

Synthetic lethality refers to the simultaneous mutation of two genes leading to cell death (Fig. 3); based on the concept of ‘synthetic lethality,’ oncologists have continuously explored cancer-targeted therapy strategies to provide innovative ideas for developing tumor drugs. Due to the diversity of the SWI/SNF complex structure, combination, and function, SWI/SNF mutations in cancer can use the synthetic lethal interaction mode to disturb other SWI/SNF subunits or some molecules related to function, highlighting their potential as drug development target molecules [75]. To date, targeted molecular inhibitors for GC, such as PARP inhibitors, PI3K / AKT / mTOR inhibitors, EZH2 inhibitors, and others, have been investigated extensively based on ARID1A gene mutation or defect combined with the concept of synthetic lethality.

#### PARP inhibitors

Inhibitors of PARP were the first synthetic lethal concept–based anticancer drugs that gained approval for clinical use. In breast cancer and ovarian cancer, tumor suppressor gene BRCA1/2 defects or mutations while inhibiting the activity of PARP can target tumor cells, leading to synthetic death [76]. In addition to BRCA1/2, ARID1A-deficient tumors also show sensitivity to PARP inhibitors [32]. However, PARP inhibitor monotherapy has minimal effect on cancers lacking ARID1A, and it often needs to work in combination with other drugs. The mechanism may be that the HR pathway in ARID1A-deficient tumors is not impaired and can repair DSB induced by replication fork stagnation and PARP [77]. In addition, it has been found that the combination of PARP inhibitor olaparib and PI3K inhibitor BKM120 may be an emerging treatment for ARID1A-deficient GC [78]. In a phase 1 clinical trial (NCT03842228), individuals with advanced solid cancers were chosen based on more than 20 gene mutations, including ARID1A, MSH2, PTEN, BARD1, BRCA1, and BRCA2. PARP inhibitors, PI3K inhibitors, and ICIs were used to evaluate treatment efficacy, but an accurate conclusion has yet to be reached.

#### Targeted PI3k/AKT/mTOR inhibitors

The PI3K/AKT/mTOR pathway regulates GC cells’ function through various mechanisms, including promoting cell invasion, metastasis, epithelial-mesenchymal transition (EMT), angiogenesis, and activating tumor chemotherapy resistance. Pan-PI3K inhibitors such as BKM120, BAY80-6946, and PI3K subunit selective inhibitors such as BYL719 and TAK117 showed sound anti-tumor effects in in vitro experiments of GC [78–83]. Still, they did not produce promising results in clinical studies. The pan-AKT inhibitor AZD5363 has been used in phase I and II clinical studies of GC with good drug resistance and safety. In addition, MK-2206, which is currently in the early clinical trial stage, is a potent and highly selective pan-AKT inhibitor [84, 85]. Traditional mTOR inhibitors that block the PI3K/AKT/mTOR signaling pathway through mTORC1, including rapamycin and its analog everolimus, have not achieved satisfactory clinical results in a phase III clinical study of everolimus as a second or third-line treatment for advanced GC [86]. The clinical effects of second- and third-generation mTOR inhibitors still require accurate reporting of extensive sample clinical data.

Although a variety of targeted molecular inhibitors can exert potential anti-tumor effects through the PI3K/AKT/mTOR pathway, to date, the clinical efficacy of these inhibitors as a monotherapy has shown meager response rates. It is possible that these failures are the result of a requirement for appropriate patient selection based on dependable biomarkers. Lee et al. [61] found that patients selected based on ARID1A expression in GC tissues had increased sensitivity to drugs that block the activity of AKT and greatly improved clinical results. A combination therapy consisting of the AKT inhibitor GSK690693 and standard chemotherapy enhances the efficacy of ARID1A knockdown GC cells and has potential in future research. A recent study showed that ARID1A could be used to screen GC patients who profit from mTOR inhibitor therapy. In terms of the mechanism, mTOR inhibitors can effectively target the activated pS6 and SOX9 in ARID1A-deficient GC [87]. In addition, Hanahan et al. [88] found that PI3K-AKT-mTOR inhibitors additionally have a significant function in regulating the tumor immune microenvironment. It has been demonstrated that inhibiting the PI3K/AKT pathway increases the sensitivity to tumor-specific CD8 + T cell-mediated cytotoxicity [89, 90]. According to other research findings, combining PI3K/AKT/mTOR inhibitors with ICIs (which include PD-1 inhibitors in parallel with CTLA-4 inhibitors) or other anti-tumor immunotherapy may enable patients to obtain the best efficacy [91].

#### EZH2 inhibitors

Recent research has revealed that numerous cancer cells exhibit EZH2 overexpression and aberrant regulation. EZH2-targeted inhibitors based on synthetic lethal effects play an essential role in ARID1A mutant cancers. The researchers found that in ovarian cancer cells with ARID1A mutation, PIK3IP1, a direct target gene of ARID1A and EZH2, was up-regulated after EZH2 was inhibited, leading to cell death by inhibiting the PI3K-AKT signaling pathway. Moreover, EZH2 inhibitor GSK126 can lead to the decline of ovarian cancer carrying ARID1A mutation in vivo [92]. Another study confirmed that EZH2 inhibitors increased the selective sensitivity of ARID1A-deficient GC [93]. In addition, inhibition of EZH2 by Tazestat or GSK126 can lead to the synthetic lethality of SMARCA4, SMARCB1, PBRM1, and other SWI/SNF chromatin remodeling complex subunit-deficient cancers [94–97]. As a popular epigenetic target, there is mounting evidence demonstrating that EZH2 can control a wide range of tumor-infiltrating lymphocytes, form an immunosuppressive microenvironment within the tumor, and allow tumor cells to escape the recognition and destruction of the immune system. Inhibition of EZH2 as an attractive therapeutic strategy can strengthen existing immunotherapy. Studies have shown that the use of EZH2 inhibitors in conjunction with ICB results in a significant degree of synergy in the treatment of specific tumors [98, 99]. Still, it must be considered that this combination therapy may lead to excessive immune system activation and increase the potential risk of autoimmune diseases. There needs to be more research on how EZH2 inhibitors and ICB work together to treat ARID1A mutant cancer.

#### Other

Other targeted drugs with an anti-tumor ability for ARID1A-deficient GC include glutathione (GSH) inhibitors, YM-155, and Nutlin-3 + TP064. Reduced solute carrier family seven-member 11(SLC7A11) recombinant protein expression decreases GSH synthesis, making ARID1A-deficient stomach cancer cells vulnerable to GSH inhibition [100]. It has been discovered that the catalytic subunit of the rate-limiting enzyme in GSH synthesis, the Glutamate-Cysteine Ligase Catalytic Subunit (GCLC), is a promising therapeutic target for tumors lacking ARID1A [101]. Therefore, developing drugs targeting the glutathione metabolic pathway may be a prospective treatment strategy for ARID1A-deficient tumors. A novel inhibitor called YM-155, which has been shown to inhibit apoptosis in vitro, targets the survivin protein. Lo et al. [102] discovered that survivin (BIRC5) might be a lethal partner for ARID1A synthesis in a genetically engineered human gastric organoid model, particularly in the early stages of ARID1A-deficient GC. Additional clinical studies are still required to understand better the connection between YM155 and malignant tumors that diminish the ARID1A gene. At the same time, the combination therapy of CARM1 inhibitor TP064, a vital regulator of the BAF complex, and P53 agonist Nutlin-3 also provides a potentially effective treatment option for GC patients with ARID1A mutation [44].

## Conclusions and prospects

**Fig. 4:**
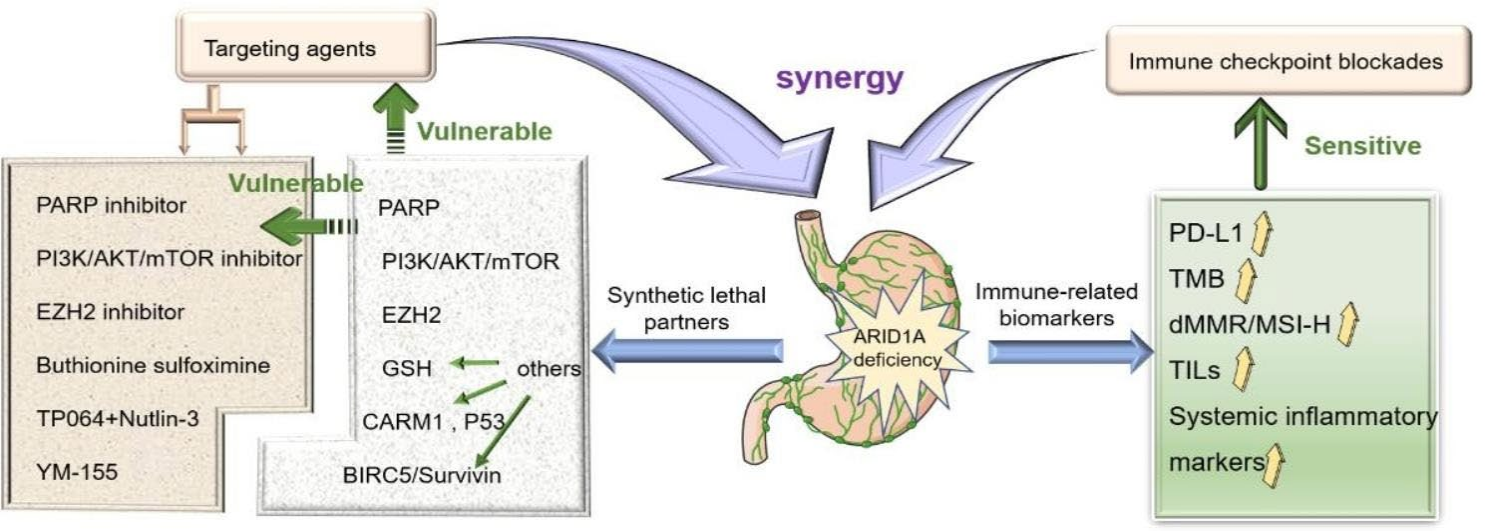
ARID1A-deficient gastric cancer immunotherapy synergistic targeted therapy strategy. ARID1A, the AT-rich interaction domain 1 A; PD-L1, programmed cell death ligand 1; TMB, tumor mutation burden; dMMR, different Mismatch Repair; MSI-H, microsatellite instability-high; TILs, Tumor Infiltrating Lymphocytes; PARP, poly (ADP-ribose) polymerase; EZH2, enhancer of zeste homolog 2; GSH, glutathione

To summarize, due to its unique advantages, the ARID1A mutation plays an essential role in the clinical practice of GC. ARID1A can be a screening biomarker for individuals whose cancer is responsive to targeted therapy and immunotherapy. Combining the two therapies may significantly increase the likelihood of successfully treating GC (Fig. 4). Studies, both preclinical and clinical, have demonstrated that the role of genetics and epigenetics in malignant tumors will be explored further, and mutation of the chromatin remodeling complex subunit, ARID1A, may become a promising tool for individualized treatment targets in patients with GC. However, some challenges need to be overcome. First, since the majority of current clinical findings were derived from a retrospective analysis of a relatively tiny sample, selection bias and confounding factors may interfere with the result. Secondly, based on the defects of single therapy resistance and combined treatment of adverse reactions, it is necessary to strictly control the dose and usage of ARID1A-mutant GC targeted therapy drugs. Third, the role of ARID1A mutation in tumorigenesis, tumor development, prediction of therapeutic response, and potential biological mechanisms needs to be further examined. Prospective clinical studies conducted across multiple centers and on a large scale are required to draw more reliable results regarding the predictive and prognostic value of ARID1A mutations in GC. On the basis of this review, we hope that the subset of patients suffering from GC with ARID1A mutation will have improved clinical outcomes.

## Data Availability

Not applicable.
